# Molten Salt Synthesis of Intermetallic Compound TiNi Nanopowder Passivated by TiO_x_ Shell Prepared from NiTiO_3_ for Catalytic Hydrogenation

**DOI:** 10.3390/ma15238536

**Published:** 2022-11-30

**Authors:** Yasukazu Kobayashi, Shota Yokoyama, Ryo Shoji

**Affiliations:** 1 Renewable Energy Research Centre, National Institute of Advanced Industrial Science and Technology, 2-2-9 Machiikedai, Koriyama 963-0298, Japan; 2 Department of Chemical Science and Engineering, National Institute of Technology, Tokyo College, 1220-2 Kunugida, Hachioji 193-0997, Japan

**Keywords:** intermetallic compound, TiNi, molten salt synthesis, 4-Nitrophenol hydrogenation

## Abstract

Titanium-nickel alloy is an attractive material due to its unique properties of shape memory effect, superior elasticity, and biocompatibility. Generally, Ti-Ni alloy powders are prepared from pure elemental powders of Ti and Ni as starting materials, but it is an energy-intensive process to obtain pure titanium. In this study, intermetallic compound TiNi powder passivated by TiO_x_ shell was prepared by directly reducing a commercial NiTiO_3_ using CaH_2_ reducing agent in a molten LiCl at 650 °C. Analyses by X-ray diffraction, scanning electron microscopy/transmission electron microscopy with energy-dispersive X-ray spectroscopy and X-ray photoelectron spectroscopy revealed that the powder had a core–shell structure, with the core of TiNi and the shell of TiO_x_-rich composition with scarce metallic Ni nicely catalyzing hydrogenation reactions with good recyclability and stability.

## 1. Introduction

Titanium-nickel alloy is an attractive material because of its unique properties of shape memory effect, superior elasticity, and biocompatibility, as well as corrosion resistance, allowing for various industrial applications in biomedical and structural engineering fields [1,2,3,4]. Ti-Ni alloy powders are typically prepared from pure elemental powders of Ti and Ni as starting materials [5,6,7,8,9,10], where they are melted at a high temperature of ~2000 °C under an inert atmosphere/vacuum for well-mixing. Some advanced methods for preparing finer ones, such as self-propagating high-temperature synthesis (SHS) [11,12,13,14,15,16,17], combustion method [18,19], plasma/laser techniques [20,21,22,23], and so on, have been reported. It is a relatively energy-intensive process to produce pure titanium, which is used as one of the raw materials to prepare titanium alloys from oxide ores, such as TiO_2_ and FeTiO_3_. Therefore, it would be innovative to prepare titanium alloys directly from titanium oxides. We previously succeeded in preparing intermetallic compound TiFe powders from the oxide precursors, including TiO_2_ [24] and FeTiO_3_ [25] by directly reducing them and then alloying them simultaneously in highly reductive conditions of molten LiCl-CaH_2_ at 600 °C. Under these conditions, hydride ions (H^−^) or calcium metals that are produced from CaH_2_ could as strong reducing agents to readily reduce the titanium oxide precursors at such a low temperature.

In this study, intermetallic compound TiNi powder was prepared by directly reducing a commercial NiTiO_3_ in molten LiCl-CaH_2_ at 650 °C. Molten salt synthesis is a good technique to obtain intermetallic compounds available for catalyst application [26]. The NiTiO_3_ precursor had a high crystallinity, indicating that Ni and Ti had previously mixed well on an atomic level, resulting in the formation of homogeneous TiNi powder by deoxidization. Nickel metals, titanium metals and their alloys are well-known active catalysts for hydrogenations [27,28,29,30]. Efficient water purification techniques are highly aspired [31,32]. In this study, the prepared TiNi was used to catalytically hydrogenate *p*-nitrophenol (4-NP) to confirm its potential application as a catalyst available in liquid phase.

## 2. Materials and Methods

Commercial NiTiO_3_ (99.9%, Kojundo Chemical Laboratory Co., Ltd., Tokyo, Japan) was used as a precursor to prepare the intermetallic compound TiNi. First, it was mixed in the air with CaH_2_ (94.0%, JUNSEI Chem. Co., Ltd., Tokyo, Japan) and LiCl (99.0%, Wako Pure Chem. Corp., Osaka, Japan) in a mortar with a weight ratio of NiTiO_3_/CaH_2_/molten salt source = 2/6/3 [33]. The mixed powder was then loaded into a stainless-steel reactor and heated in argon for 2 h at 650 °C. The reduction temperatures of 650 °C were chosen because the intermetallic TiNi phase is stable above 630 °C [34]. Finally, the reduced precursors were crushed in a mortar and rinsed with a 0.1 M NH_4_Cl aqueous solution made with NH_4_Cl (99.5%, Wako Pure Chem. Corp.) and distilled water to obtain the final product powder (TiNi).

The crystal structure of the prepared samples was examined using X-ray diffraction (XRD, MiniFlex 600, Rigaku, Tokyo, Japan) with CuK_α_ radiation at 40 kV and 15 mA. The measurements were ranged from 20° to 130° with a step interval of 0.01° and a scan speed of 10°/min. The porosity was examined using N_2_ adsorption at −196 °C (BELLSORP mini-II, MicrotracBEL Corp., Osaka, Japan). The samples were pretreated at 200 °C for 30 min under a vacuum in order to remove the water contained in the samples before the measurement. Scanning electron microscopy (SEM, JSM-7400F, JEOL Ltd., Tokyo, Japan) and transmission electron microscopy (TEM, a Tecnai Osiris, FEI system) were used to examine the morphology, and elemental analysis was performed using energy dispersive X-ray spectrometry (EDX). Cupper-based micro grids (NP-C15 (Lacy Carbon film), Okenshoji Co., Ltd., Tokyo, Japan) were used to fix the sample powder and therefore non-identified signals in the images are mainly due to the cupper. The chemical states and composition of the prepared samples’ surface were determined using X-ray photoelectron spectroscopy (XPS) (PHI X-tool, ULVAC-PHI, Inc., Kanagawa, Japan) operated with AlK_α_ radiation. The chemical shifts were calibrated by fixing C1s peak of the surface carbonaceous contaminants at 284.8 eV.

The catalytic reactions were conducted in 20 mL glass bottles following the previously reported procedures [35]. In the catalytic tests, 1 mL of 4-NP solution (14 mM) was added to a bottle containing 10 mg of catalyst powder, 1 mL of NaBH_4_ solution (0.42 M), and 7 mL of distilled water as the solvent. To satisfy first-order reaction kinetics, the initial concentration of NaBH_4_ (0.047 M) was 30 times higher than that of 4-NP (1.6 mM). The reactions were stirred at 50 °C until the concentrations reached zero. An aluminum heat sink mounted on a hotplate was used to maintain a constant solution temperature. A small aliquot (100 μL) solution was taken to determine the concentration changes at reaction times of 0.5–50 min. The conversion of 4-NP to *p*-aminophenol was monitored using an ultraviolet-visible spectrometer using the respective absorbance changes at 401 and 315 nm. NiTiO_3_ and TiO_2_ (JRC-TIO-4 (2) (Degussa P25), supplied by Japan Reference Catalyst Society, 50 m^2^/g [36]) were also tested for references.

## 3. Results and Discussion

### 3.1. Synthesis of TiNi Nanopowder from NiTiO_3_

**Figure 1:**
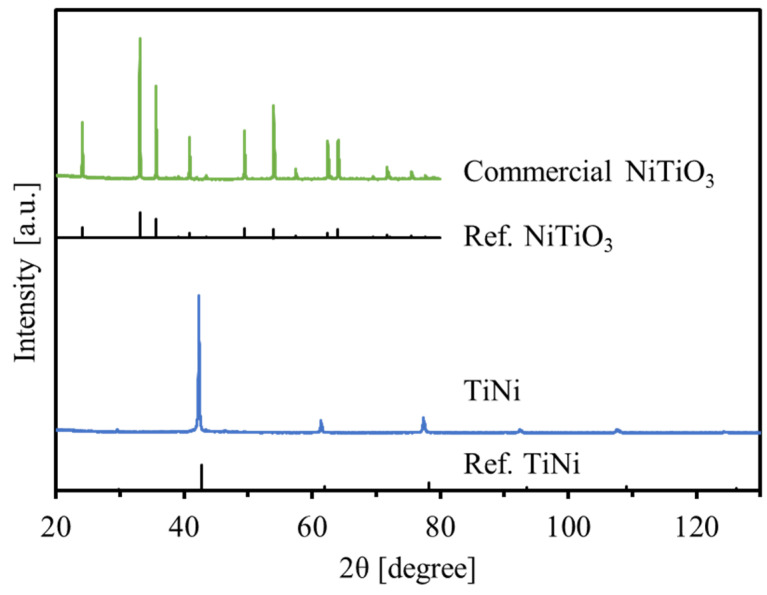
XRD patterns of commercial NiTiO_3_ and the prepared TiNi.

Figure 1 shows the XRD patterns of commercial NiTiO_3_ and prepared TiNi. The observed peaks were perfectly identified as NiTiO_3_ and intermetallic compound TiNi with a cubic CsCl-type crystal structure, respectively. The crystallite sizes were calculated using the Sherrer equation with the main peak observed around 42°–43° for the intermetallic TiNi was 72.9 nm. The measured BET surface area using N_2_ adsorption was 6.0 m^2^/g. These values are summarized in Table 1. A larger crystallite size TiNi with a smaller BET surface area was obtained in this study compared with our previous results of TiFe (46.2–65.2 nm, 13.9–20.0 m^2^/g) [24,25]. Because nickel oxides are more easily reduced than iron oxide because of the more thermodynamic stability of FeO than NiO [37], the rate of crystal growth for TiNi crystal particles in the reduction/alloying processes at 650 °C could be accelerated to form the final larger particles.

**Figure 2:**
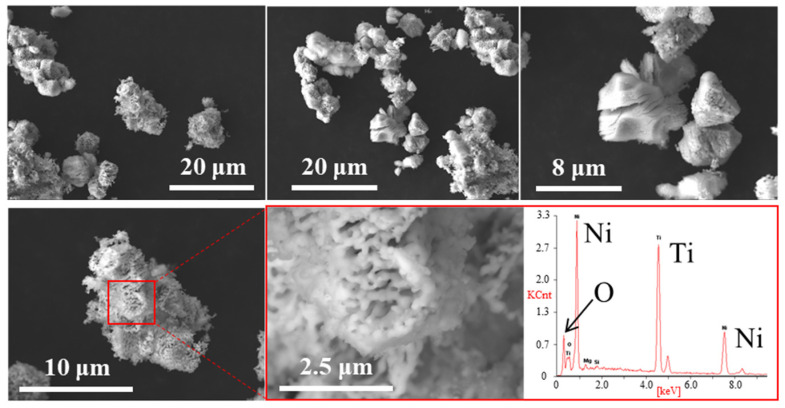
SEM images for the prepared TiNi. EDX spectrum is given at the right-down with Ti/Ni/O = 40.4/42.8/16.8 [mol%].

**Figure 3:**
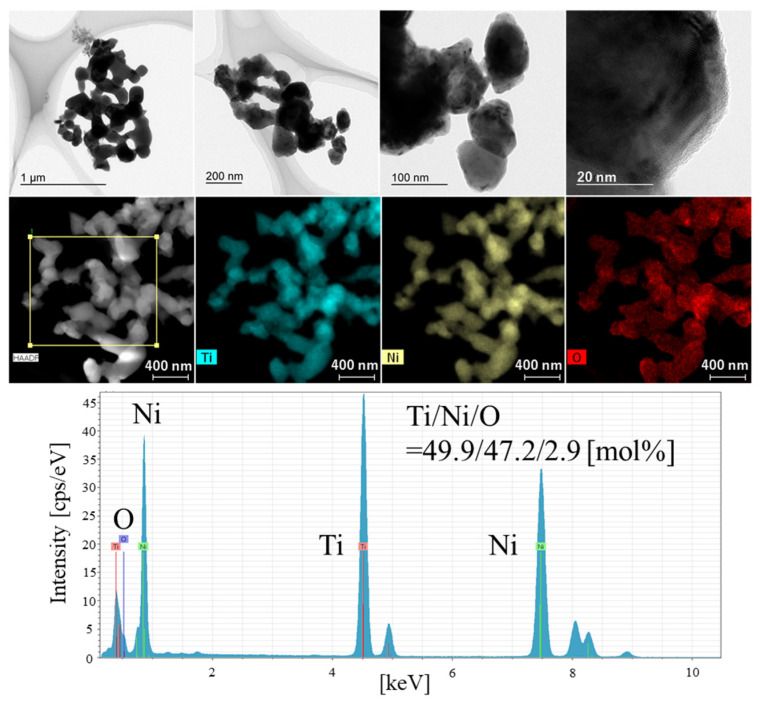
TEM images and the EDX result for the prepared TiNi.

Figure 2 and Figure S1 show the SEM images for NiTiO_3_ and the prepared TiNi, respectively. For NiTiO_3_, rocky pieces with high crystallinity were observed. Smooth surfaces with microscale morphologies were observed in magnified views. Microscale TiNi pieces were similarly observed, but when examined under high magnification, their porosity was very high in the nanoscale range. In the deoxidization process where oxygen atoms were removed from NiTiO_3_ to form TiNi, the aggregation of previously oxygen-occupied spaces in the final TiNi could attribute to the formation of the porous structure. Similar nanosized morphologies were also observed in the TiNi powder prepared similarly but at 800 °C (Figure S2), indicating that the fine morphologies in the prepared TiNi may be thermally stable. Elemental analysis by SEM-EDX was also performed on the prepared TiNi (Figure 2). Impurity elements were detected in small amounts except for Ti, Ni, and O. The molar ratio of Ti/Ni/O was 40.4/42.8/16.8 (Table 1). The result confirmed the formation of the intermetallic compound TiNi with a stoichiometric molar ratio of 1 to 1. Note that the oxygen ratio was a little bit high, indicating surface oxidation (which is discussed later). TEM-EDX was used on the prepared TiNi to examine finer morphology with elemental analysis. EDX was used to perform the elemental analyses at two different positions (Figure 3 and Figure S3). The observed elemental molar ratios are summarized in Table 1. Nanoscale particles nearly corresponding to the crystalline size (72.9 nm) as determined by XRD, were observed in the TEM images. The elemental mappings of Ti and Ni overlapped well, and the molar ratios of the intermetallic compound TiNi were almost stoichiometric. These results also demonstrate the successful preparation of the intermetallic compound TiNi by the direct reduction of NiTiO_3_.

**Figure 4:**
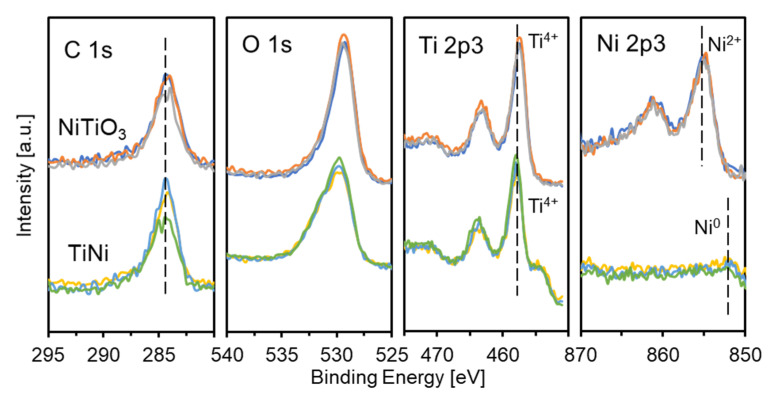
XPS spectra for NiTiO_3_ (top) and the prepared TiNi (bottom). Each data was measured at three different positions, and each data is shown above by different color.

To examine the surface chemical states, XPS measurements were performed on NiTiO_3_ and the prepared TiNi. The analyses were performed at three different positions for each sample to guarantee the measurement errors. The obtained spectra for C 1s, O 1s, Ti 2p3, and Ni 2p3 orbitals and the molar ratios of Ti/Ni/O are described in Figure 4 and Table 1, respectively. The XPS spectra measured in a wide-scanned mode are shown in Figure S4, where the other elements except Ti, Ni, and O were barely detected. For the O 1s orbital, large signals in a similar degree with NiTiO_3_ were observed for TiNi. The oxygen ratios determined by XPS were much higher than those determined by SEM-/TEM-EDX (Table 1). These results indicated that the surface of the prepared TiNi powder was in the form of oxides. For the Ti 2p3 orbital, clear signals assigned to Ti (+4) [38] were observed for both NiTiO_3_ and TiNi, indicating the formation of TiO_x_ species on the prepared TiNi surface. For the Ni 2p3 orbital, very small signals assigned to Ni (0) [39] were observed for TiNi, whereas distinct signals to Ni (+2) [39] were observed for NiTiO_3_. Ti-rich/Ni-deficient surface compositions by XPS for TiNi (Table 1), were extremely different from those by SEM-/TEM-EDX. It was speculated that the prepared TiNi powder has too thin TiO_x_ surface layers to observe via the employed TEM. Taking the results of XRD, SEM-/TEM-EDX, and XPS together into account, the obtained TiNi possessed a core–shelled structure, such as the core of the intermetallic compound TiNi and the shell of TiO_x_-rich composition with a trace of metallic Ni.

### 3.2. Catalytic Hydrogenation of 4-Nitrophenol

**Figure 5:**
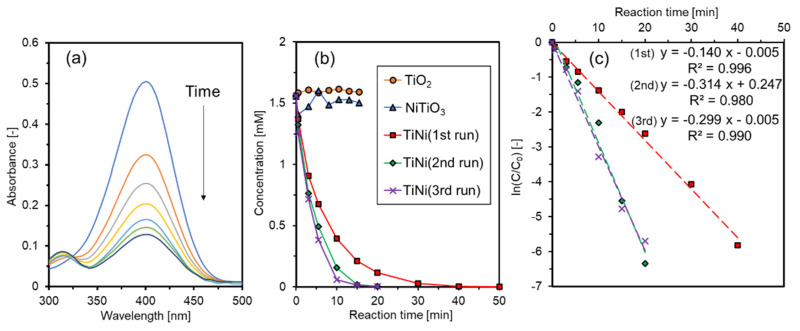
(**a**) Absorbance changes of 4-NP solutions with a reaction time for the prepared NiTi, (**b**) changes in 4-NP concentration (C) versus time for the TiNi at 50 °C, NiTiO_3_ and TiO_2_ at 40 °C, and (**c**) a plot of ln(C/C_0_) versus time to acquire rate constants (k). For the prepared TiNi, the catalytic tests were repeated three times to examine the recyclability.

**Figure 6:**
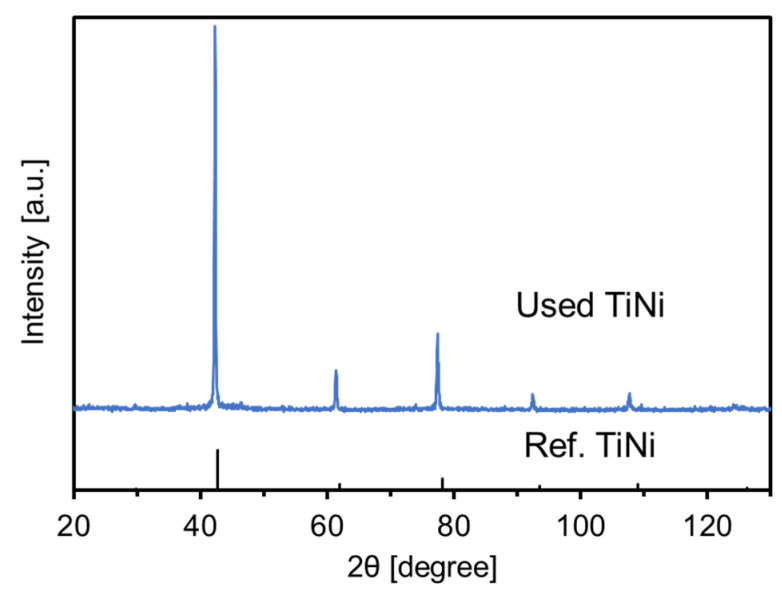
XRD patterns of the used TiNi catalyst powder.

The morphology of the catalyst plays an important role for the catalytic activity. Currently, some core–shelled and hollow structures are extensively used in catalysis, showing unique and superior catalytic performance [40,41,42]. In this study, we evaluated the catalytic performance of the core–shelled TiNi in hydrogenation of 4-NP to 4-aminophnol (4-AP). NaBH_4_ was used to catalyze the hydrogenation of 4-NP at 40–50 °C using the prepared TiNi, NiTiO_3_, and TiO_2_. The absorbance change in the reaction solution during the TiNi reaction is shown in Figure 5a. The absorbance at 315 nm to the production concentration of *p*-aminophenol increased, as the absorbance at 401 nm corresponding to the concentration of 4-NP decreased with reaction time, indicating the progress of the hydrogenation reaction. The concentration change in 4-NP during the reactions with catalysts TiNi, NiTiO_3_, and TiO_2_ is shown in Figure 5b. For NiTiO_3_ and TiO_2_ at 40 °C, little changes were observed in the concentrations, indicating that they had no catalytic abilities in the reaction system. For TiNi at 50 °C, the catalytic tests were conducted there times repeatedly with the identical catalyst powder in order to confirm the recyclability of the prepared TiNi powder. The concentration decreased rapidly with the reaction time and 100% conversion was obtained in 15–40 min. These results showed that the active species were not TiO_x_ and Ni (+4) but metallic Ni (0). The activities of 2nd and 3rd runs were higher than 1st one. This could be because the TiNi catalyst was more activated in the 2nd and 3rd runs due to the removal of surface TiO_x_ passivation during the hydrogenation reaction. The reaction rate constant of 0.14–0.31 min^−1^ was obtained from a plot of ln(C/C_0_) versus time (Figure 5c), where C and C_0_ represent the concentration of 4-NP at distinct and initial times, respectively. The rate constants of this study and previous studies with nickel-based catalysts are summarized in Table 2. Because the reaction conditions differed, a quantitative comparison was difficult. Our result was reasonably comparable with those obtained using the previously reported nickel-based catalysts. Particularly, the prepared TiNi exhibited a higher constant than the multicomponent alloy catalysts of AlCoCrFeNiV and CrMnFeCoNi under similar reaction conditions. Despite a limited amount of metallic Ni (0) exposed on the surface, as confirmed by XPS, the prepared TiNi exhibited promising catalytic performance. As the results of the previous works [43,44], heterogeneous hydrogenation of 4-NP to 4-AP by NaBH_4_ proceeds in accordance with the Langmuir–Hinshelwood (LH) model. In the first step, the NaBH_4_ is decomposed by hydrolysis, then the B(OH)_4_^−^ and active hydrogen (or, hydride) are formed. The active hydrogen diffuses to adsorb on the surface of active metals, such as gold and silver nanoparticles. 4-NP also diffuses to adsorb on the surface. Finally, the adsorbed active hydrogen reacts with 4-NP to yield the product 4-AP. The rate-determining step is given by the surface reaction of the adsorbed species. In our study, therefore, the surface-exposed Ni (0) was well-dispersed across the surface in the form of small nanoparticles, resulting in numerous active sites. The active sites could effectively work to promote the surface reaction to obtain the fast reaction rates. The XRD patterns of the used TiNi catalyst powder is shown in Figure 6. The identical XRD peaks with the fresh TiNi powder were observed and the result indicated that the prepared TiNi powder was stable without decomposition during the hydrogenation reactions.

## 4. Conclusions

Intermetallic compound TiNi powder passivated by TiO_x_ shell was successfully prepared from NiTiO_3_ by reducing it at 650 °C in a molten LiCl-CaH_2_ system. XRD, SEM-/TEM-EDX, and XPS analyses demonstrated that the obtained TiNi possessed a core–shelled structure, such as the core of intermetallic compound TiNi and the shell of TiO_x_-rich composition with a scarce amount of metallic Ni. The scarce metallic Ni demonstrated promising high catalytic performance in the hydrogenation of 4-NP because of the good dispersion of active Ni species on the surface.

## Figures and Tables

**Table 1:** Crystalline sizes, BET surface areas (S.A.), and elemental molar ratios measured by XPS, SEM-EDX, and TEM-EDX.

Sample	Crystalline Size [nm]	BET S.A. [m^2^/g]	Elemental Molar Ratio [mol%]			
			Method	Ti	Ni	O
TiNi	72.9	6.0	XPS	23.6	1.1	75.2
				24.3	1.0	74.7
				24.7	2.0	73.3
			SEM-EDX	40.4	42.8	16.8
			TEM-EDX	49.9	47.2	2.9
				49.9	44.4	5.7

**Table 2:** Comparison of rate constants (k) for 4-NP reduction.

Sample	Temp. [°C]	Reaction Conditions	k [min^−1^]	Ref.
TiNi	50	4-NP (1.6 mM) NaBH_4_ (47 mM) 10 mg-cat/9 mL	0.14–0.31	This study
Ni film	25	4-NP (0.1 mM) NaBH_4_ (10 mM) 15 cm^2^-cat/16 mL	0.09	[45]
Co_50_Ni_50_ film			0.15	
Co_25_Ni_75_ film			0.14	
p(MAc)-Ni	30	4-NP (10 mM) NaBH_4_ (400 mM) 5 mg-cat(Ni)/50 mL	0.75	[46]
Ni-RGO	R.T.	4-NP (0.1 mM) NaBH_4_ (30 mM) 10 mg-cat/104 mL	0.07	[47]
Ni NP			0.02	
SiO_2_@C/Ni	R.T.	4-NP (0.2 mM) NaBH_4_ (65 mM) 3 mg-cat/3.1 mL	2.19–3.06	[48]
AlCoCrFeNiV	53	4-NP (1.6 mM) NaBH_4_ (47 mM) 10 mg-cat/9 mL	0.05	[49]
CrMnFeCoNi	50	4-NP (0.16 mM) NaBH_4_ (60 mM) 10 mg-cat/9 mL	0.11	[35]

## Data Availability

Not applicable.

## Supplementary Materials

The following supporting information can be downloaded at: https://www.mdpi.com/article/10.3390/ma15238536/s1, Figure S1. SEM images of commercial NiTiO_3_. Figure S2. SEM images of TiNi prepared by reducing NiTiO_3_ via CaH_2_ in molten LiCl at 800 °C. Figure S3. TEM images and the EDX result of the prepared TiNi. Figure S4. XPS spectra measured in a wide-scan mode of NiTiO_3_ and the prepared TiNi.
