# Supplementary material for: Molten Salt Synthesis of Intermetallic Compound TiNi Nanopowder Passivated by TiOx Shell Prepared from NiTiO3 for Catalytic Hydrogenation
Source: Materials (Basel). 2022 Nov 30;15(23):8536. doi: 10.3390/ma15238536 (PMC9736321; doi:10.3390/ma15238536)
Supplement: Supplementary file 1 [file materials-15-08536-s001.zip › materials-2008724-supplementary.pdf]

Communication

Molten Salt Synthesis of Intermetallic Compound TiNi Nanopowder Passivated by TiO_x Shell Prepared from $NiTiO_3$ for Catalytic Hydrogenation

Yasukazu Kobayashi [1,*], Shota Yokoyama [2] and Ryo Shoji [2]

[1] Renewable Energy Research Centre, National Institute of Advanced Industrial Science and Technology, 2-2-9 Machiikedai, Koriyama 963-0298, Japan
[2] Department of Chemical Science and Engineering, National Institute of Technology, Tokyo College, 1220-2 Kunugida, Hachioji 193-0997, Japan
* Correspondence: yasu-kobayashi@aist.go.jp; Tel.: +8129-861-4014

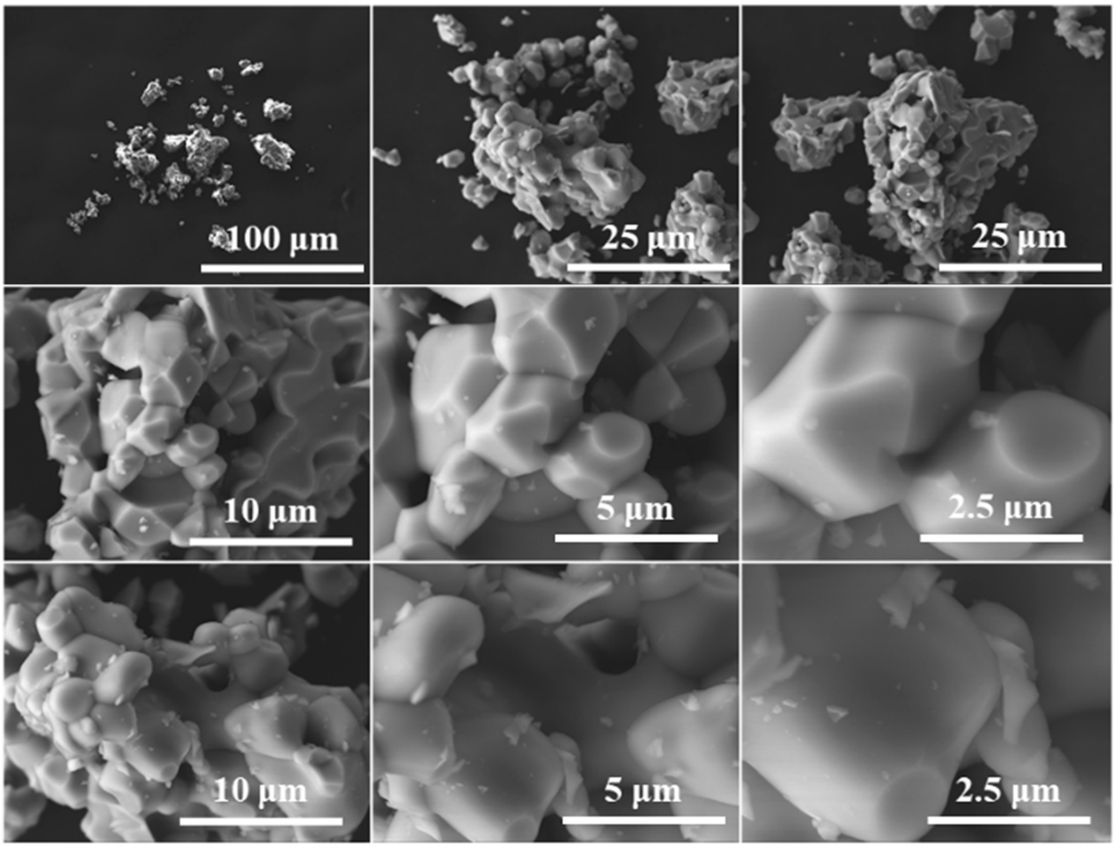

Fig. S1. SEM images of commercial $NiTiO_3$.

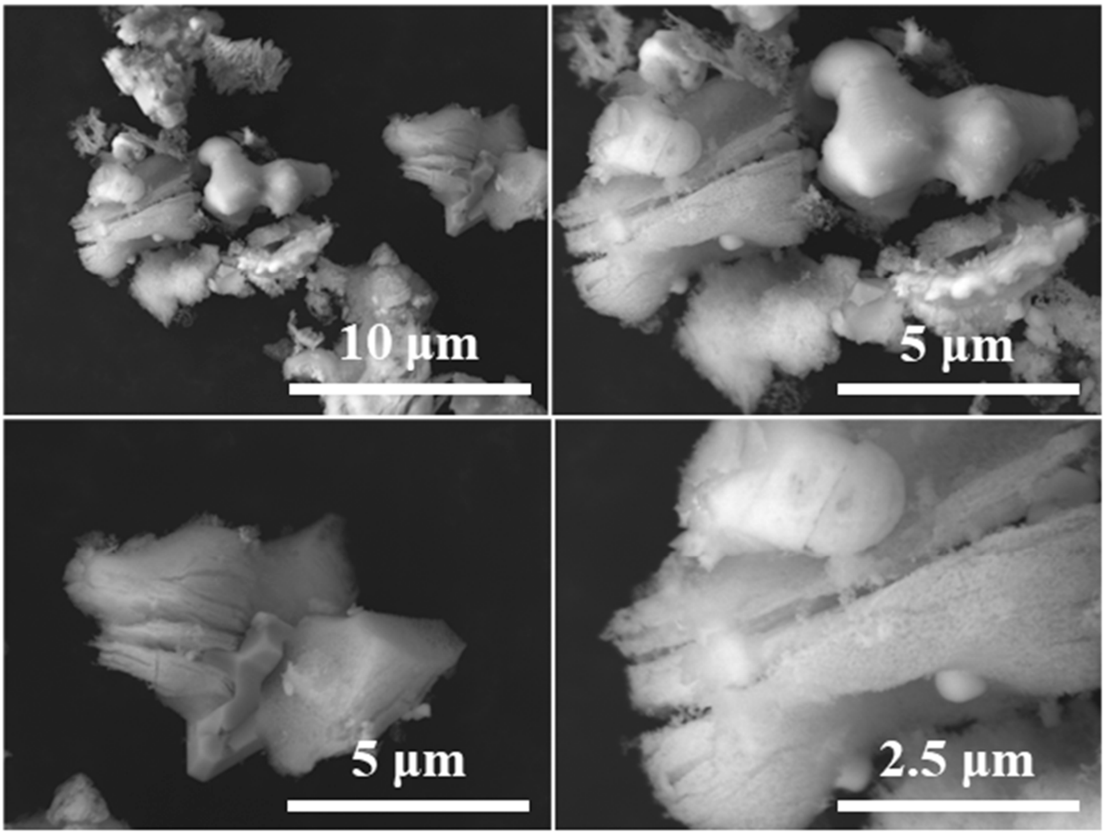

Fig. S2. SEM images of TiNi prepared by reducing $NiTiO_3$ via CaH_2 in molten LiCl at 800°C.

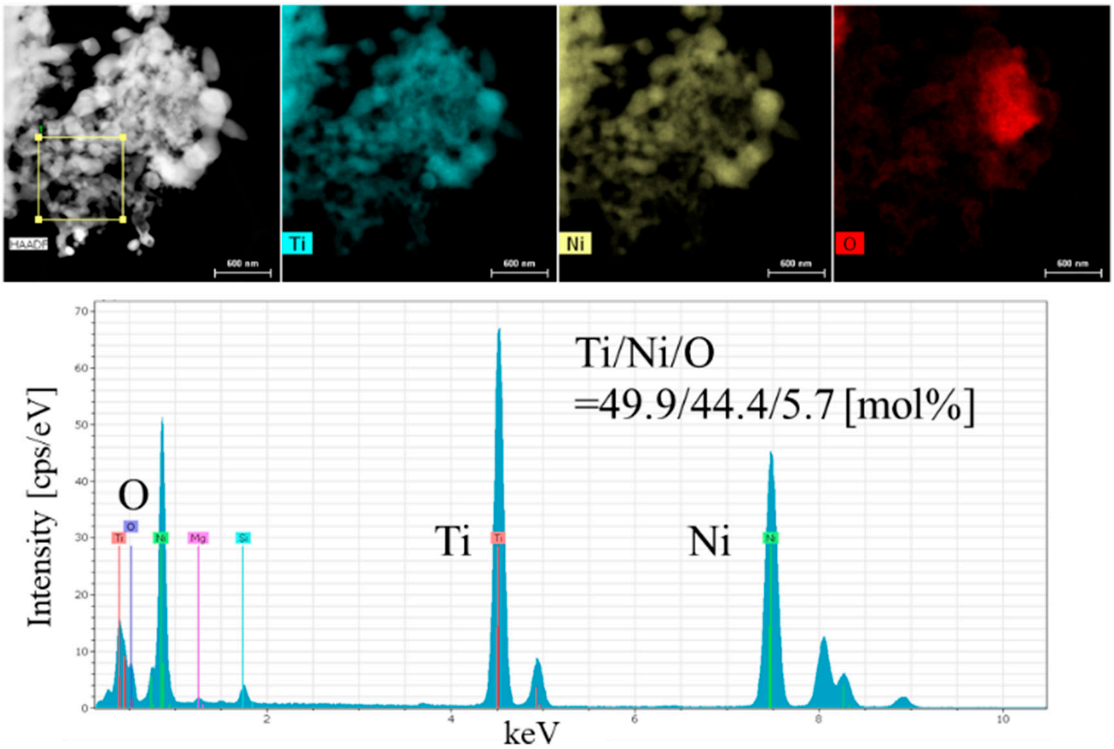

Fig. S3. TEM images and the EDX result of the prepared TiNi.

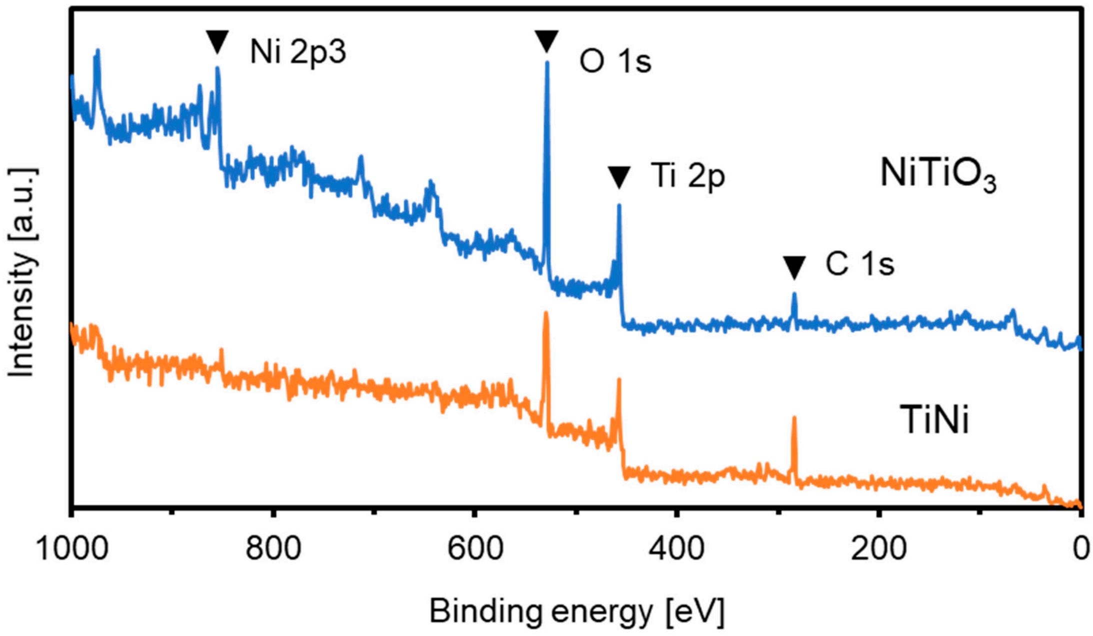

Fig. S4. XPS spectra measured in a wide-scan mode of $NiTiO_3$ and the prepared TiNi.
